# Hearing and vision impairment and social isolation over 8 years in community-dwelling older adults

**DOI:** 10.1186/s12889-024-17730-8

**Published:** 2024-03-13

**Authors:** Alison R. Huang, Thomas K. M. Cudjoe, George W. Rebok, Bonnielin K. Swenor, Jennifer A. Deal

**Affiliations:** 1 Department of Epidemiology, Johns Hopkins Bloomberg School of Public Health, Baltimore, MD 21202 USA; 2 Cochlear Center for Hearing and Public Health, Johns Hopkins Bloomberg School of Public Health, Baltimore, MD USA; 3 Division of Geriatric Medicine and Gerontology, Johns Hopkins School of Medicine, Baltimore, MD USA; 4 Department of Mental Health, Johns Hopkins Bloomberg School of Public Health, Baltimore, MD USA; 5 Department of Otolaryngology-Head and Neck Surgery, Johns Hopkins School of Medicine, Baltimore, MD USA; 6 Disability Health Research Center, Johns Hopkins Bloomberg University, Baltimore, MD USA; 7 The Wilmer Eye Institute, Johns Hopkins University, Baltimore, MD USA; 8 Johns Hopkins School of Nursing, Baltimore, MD USA

**Keywords:** Hearing impairment, Vision impairment, Social isolation, Longitudinal

## Abstract

**Background:**

Little is known about the long-term impact of hearing and vision impairment on social isolation. This study quantifies the association between hearing, vision, and concurrent hearing and vision impairment (dual sensory impairment) and social isolation over 8 years among older adults.

**Methods:**

Data were from the National Health and Aging Trends Study (NHATS), a cohort study (2011 – 2019) of U.S. Medicare beneficiaries aged 65 years and older. Social isolation was measured by a binary indicator incorporating four domains: living arrangement, core discussion network size, religious attendance, and social participation. Hearing, vision, and dual sensory impairments were measured by self-report and modeled categorically (no impairment [ref.], hearing impairment only, vision impairment only, dual sensory impairment). Associations between sensory impairments and odds of social isolation over 8 years were assessed using multivariate generalized logistic mixed models and adjusted for demographic and health characteristics.

**Results:**

Among 5,552 participants, 18.9% self-reported hearing impairment, 4.8% self-reported vision impairment, and 2.3% self-reported dual sensory impairment. Over 8 years, hearing impairment only was associated with 28% greater odds of social isolation. Participants with hearing impairment only were more likely to live alone and have limited social participation.

**Conclusion:**

Greater clinical awareness of hearing impairment as a risk factor for social isolation can increase opportunities to identify and aid older adults who may benefit from resources and interventions to increase social connection and mitigate social isolation.

**Supplementary Information:**

The online version contains supplementary material available at 10.1186/s12889-024-17730-8.

## Introduction

Social isolation is an “objective state of having few social relationships or infrequent social contact with others.” [1] Social isolation can lead to harmful health outcomes (e.g., dementia, depression, and lower quality of life) and can also limit access to health promoting resources (e.g., shared information, emotional support, physical assistance) potentially available through a social support network. [1–4] In the United States (U.S.), 24% of older adults are socially isolated. [5].

Social isolation is most common among older adults who are unmarried, male, and/or have lower income and education [5]. Sensory impairments, particularly hearing and vision impairments, have also been linked to social isolation [6–12]. Sensory impairment may impact daily functioning, mobility, and ability to communicate, which may disrupt the quantity and quality of one’s social interactions as well as the ability to participate in activities that promote socialization [13–15]. The association between sensory impairment and social isolation in older adults has been investigated to some extent; however, gaps in the literature remain.

Notably, previous cross-sectional studies have demonstrated higher prevalent social isolation and restriction of social activities among older adults with hearing or vision impairment [6–10, 12]; however, evidence regarding the longitudinal impact of sensory impairment on social isolation over time is limited [16, 17]. Additionally, little is known about concurrent hearing and vision impairment (dual sensory impairment) and social isolation. Older adults with dual sensory impairment may be especially at risk for social isolation as their ability to compensate for loss of function in one sense with another sense is limited. Thus, communicating with others and engaging in social activities can be even more challenging for older adults with dual or multiple sensory impairments compared to those with a single or no sensory impairment [18].

Furthermore, examining self-reported sensory impairment, a construct distinct from objective sensory impairment that considers the perspective of functional disability is important for fully describing how sensory impairment impacts social isolation. This construct incorporates facets of sensory impairment that are not captured by objective measures yet independently contribute to the impact of sensory impairment on health [19, 20] and are critical for providing a more comprehensive understanding of sensory impairment.

Examining the connection between hearing, vision, and dual sensory impairment and social isolation may yield informative insights for addressing social isolation. Given older adults, and particularly older adults with sensory loss, have more frequent health care encounters [21, 22], there is significant opportunity within the health care system for identifying and mitigating social isolation with potential beneficial effects on associated downstream health and mental health outcomes.

In a nationally representative sample of community-dwelling older adults in the U.S., we examine the associations between self-reported hearing, vision, and dual sensory impairment and social isolation over 8 years. We examine associations with overall social isolation and with four specific domains to assess social isolation (living arrangement, core discussion network size, religious services attendance, and social participation). This study adds to the limited literature on sensory impairment and social isolation in older adults.

## Methods

### Participants

Data for this study come from The National Health and Aging Trends Study (NHATS). NHATS is a nationally representative, longitudinal study of Medicare beneficiaries in the U.S. over age 65 [23]. This study uses data collected between 2011 – 2019. Data were collected in-person. The analytic sample excluded participants classified as having possible or probable dementia and those who did not have data on cognitive status at baseline (*n* = 2,670). Cognitive impairment may impact how participants answer questions about their sensory impairment and level of social isolation. Participants with missing hearing and vision impairment data at baseline (*n* = 23) were also excluded. The analytic sample includes 5,552 participants who were cognitively normal and had complete data on sensory impairment at baseline. This study used publicly available, non-identifiable data and was approved by the John Hopkins Bloomberg School of Public Health Institutional Review Board. Informed consent was obtained by NHATS investigators from all participants.

### Measures

#### Social isolation

Social isolation was measured as a binary indicator of social isolation typology constructed by Cudjoe et al. [5]. Participants received one point for each of the following: live with at least one other person, have two or more people to talk about “important matters” with, past month attendance at religious services, and past month participation in other activities, such as clubs, meetings, or volunteer work. Participants with a score of 0 or 1 were considered “socially isolated.” The four domains included in the typology (living arrangement, core discussion network size, religious services attendance, and social participation) were also assessed as independent outcomes.

#### Sensory impairment

Sensory impairment was measured by a series of questions about ability to hear and see in certain situations [24]. Participants were instructed to include sensory ability when using hearing aids and/or glasses and contacts (if applicable) when responding. Participants were considered to have hearing impairment if they reported any of the following: 1) deafness, 2) hearing aid use, 3) unable to hear well enough to use the telephone, or 4) unable to hear well enough to carry on conversation in a room with the TV or radio playing. Participants were considered to have vision impairment if they reported any of the following: 1) blindness, 2) unable see well enough to recognize someone across the street, or 3) unable to see well enough to read newspaper print. We modeled hearing and vision impairment together in a 4-level categorical variable to measure dual sensory impairment (no impairment [reference], hearing impairment only, vision impairment only, dual sensory impairment [hearing and vision impairment]).

#### Covariates

Covariates (measured at baseline) included age (65–69, 70–74, 75–79, 80–84, 85–89, 90 +), sex (male/female), education (less than high school, high school, and greater than high school), race/ethnicity (non-Hispanic White, non-Hispanic Black, Hispanic, and Other), smoking (ever-smoker/non-smoker), and self-reported history (yes/no) of diabetes, hypertension, heart attack, heart disease, lung disease, cancer, or stroke.

### Statistical analysis

The distribution (frequency (proportion)) of baseline participant characteristics was calculated by sensory impairment status. Using generalized logistic mixed models, longitudinal models separately assessed 8-year associations between sensory impairment (no impairment [reference], hearing impairment only, vision impairment only, dual sensory impairment [hearing and vision impairment]) and overall social isolation as well as between sensory impairment and the four specific domains (living arrangement, core discussion network size, religious services attendance, and social participation). Generalized estimating equations (GEE) are useful for estimating population-level average effects and for assessing change in binary outcomes over time. An unstructured covariance matrix was conservatively assumed with robust variance estimate. All models were adjusted for age, sex, education, race/ethnicity, smoking status, hypertension, diabetes, stroke, heart attack, heart disease, lung disease, and cancer.

In sensitivity analyses, we tested sensitivity of our findings to potential non-linear change in social isolation over time. We estimated the primary model using a linear spline for time with one knot at round 5 to allow for separate estimation of the associations between hearing, vision, and dual sensory impairment (vs. no impairment) and odds of social isolation over time prior to and after round 5. We also separately assessed the associations between a) hearing impairment (vs. no hearing impairment) and b) vision impairment (vs. no vision impairment) and the odds of social isolation over time.

## Results

Of 5,552 participants, 18.9% self-reported hearing impairment only, 4.8% self-reported vision impairment only, and 2.3% self-reported dual sensory impairment at baseline (Table 1). Participants with hearing, vision, and dual sensory impairment (vs. no impairment) were more likely to be older (90 years and over: 11.0%, 7.2%, 22.8%, respectively vs. 2.8%) and have less than a high school education (20.3%, 28.8%, 42.1%, respectively vs. 19.1%). Men were more likely to have hearing impairment only (54.1%) while women were more likely to have vision and dual sensory impairments (72.7% and 59.1% respectively). Compared to participants with no impairment, hearing impairment only was more common among White participants (84.4% vs. 70.1%). Vision impairment only was more common among Black participants (28.0% vs. 22.2%), and dual sensory impairment was more common among Hispanic participants (13.4% vs. 4.5%). Participants with hearing, vision, and dual sensory impairment were more likely to report history of chronic conditions compared to participants with no impairment: heart attack (19.0%, 17.6%, 18.9%, respectively vs. 11.6%), heart disease (21.8%, 26.1%, 25.2% respectively vs. 15.1%), lung disease (16.8%, 22.0%, 15.0%, respectively vs. 14.4%), and stroke (10.9%, 19.3%, 13.5%, respectively vs. 7.9%).

**Table 1:** Participant characteristics by functional sensory impairment status; National Health and Aging Trends Study, 2011–2019

	No Impairment *N* = 4111	Hearing Impairment Only *N* = 1050	Vision Impairment Only *N* = 264	Dual Sensory Impairment *N *= 127
Age group (years), N(%)				
65–69	1074 (26.1)	112 (10.7)	62 (23.5)	12 (9.4)
70–74	1065 (25.9)	190 (18.1)	51 (19.3)	15 (11.8)
75–79	870 (21.2)	216 (20.6)	48 (18.2)	17 (13.4)
80–84	694 (16.9)	236 (22.5)	52 (19.7)	26 (20.5)
85–89	291 (7.1)	180 (17.1)	32 (12.1)	28 (22.0)
90 +	117 (2.8)	116 (11.0)	19 (7.2)	29 (22.8)
Sex, N(%)				
Male	1620 (39.4)	568 (54.1)	72 (27.3)	52 (40.9)
Female	2491 (60.6)	482 (45.9)	192 (72.7)	75 (59.1)
Race, N(%)				
Non-Hispanic White	2880 (70.1)	886 (84.4)	163 (61.7)	92 (72.4)
Non-Hispanic Black	911 (22.2)	88 (8.4)	74 (28.0)	13 (10.2)
Hispanic	187 (4.5)	36 (3.4)	17 (6.4)	17 (13.4)
Other	133 (3.2)	40 (3.8)	10 (3.8)	5 (3.9)
Education, N(%)				
Less than high school	777 (19.1)	211 (20.3)	76 (28.8)	53 (42.1)
High school	1470 (36.0)	390 (37.6)	99 (37.5)	41 (32.5)
More than high school	1831 (44.9)	437 (42.1)	89 (33.7)	32 (25.4)
Diabetes, N(%)	955 (23.2)	242 (23.0)	100 (37.9)	33 (26.0)
Hypertension, N(%)	2738 (66.7)	683 (65.1)	201 (76.1)	91 (71.7)
History of Heart Attack, N(%)	478 (11.6)	199 (19.0)	46 (17.6)	24 (18.9)
Heart Disease, N(%)	619 (15.1)	229 (21.8)	69 (26.1)	32 (25.2)
Lung Disease, N(%)	590 (14.4)	176 (16.8)	58 (22.0)	19 (15.0)
History of Cancer, N(%)	1032 (25.1)	328 (31.2)	66 (25.0)	32 (25.2)
History of Stroke, N(%)	324 (7.9)	114 (10.9)	51 (19.3)	17 (13.5)
Ever Smoker, N(%)	2134 (51.9)	556 (53.0)	136 (51.7)	61 (48.0)
Socially isolated, (N%)	905 (22.4)	71 (28.1)	242 (23.6)	40 (33.9)
Live alone, (N%)	1258 (30.8)	111 (42.0)	343 (32.8)	51 (40.2)
Small core discussion network size, (N%)	1839 (45.4)	120 (47.4)	485 (47.1)	57 (48.3)
No past month religious services attendance, (N%)	1586 (38.6)	102 (38.6)	417 (39.7)	60 (47.2)
No past month social participation, (N%)	2087 (50.8)	150 (56.8)	530 (50.5)	85 (66.9)

**Fig. 1:**
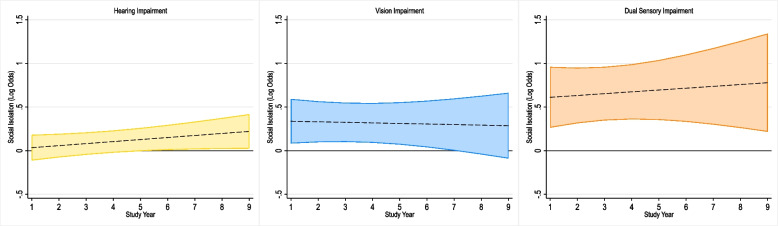
Legend: Multivariate linear mixed-effects models for adjusted odds of social isolation by study year and functional hearing impairment status, functional vision impairment status, and functional dual sensory impairment status; National Health and Aging Trends Study, 2011–2019 Models adjusted for age, sex, education, and race/ethnicity, smoking status, hypertension, diabetes, stroke, heart attack, heart disease, lung disease, and cancer

In the social isolation model (Table 2, Fig. 1), hearing impairment only (vs. no impairment) was associated with lower odds of overall social isolation at baseline (OR: 0.79; 95% CI: 0.67, 0.92). Over 8 years, hearing impairment only (vs. no impairment) was associated with 28% higher odds of social isolation (OR: 1.28; 95% CI: 1.01, 1.62). No significant differences were observed by vision or dual sensory impairment status; however, the magnitude of association for change in social isolation over time for dual sensory impairment (OR: 1.22; 95% CI: 0.62, 2.40) was nearly the same as observed for hearing impairment only (OR: 1.28; 95% CI: 1.01, 1.62), but confidence intervals were wider potentially due to lower sample size (vison impairment: *n* = 264, dual sensory impairment: *n* = 127).

**Table 2:** Adjusted baseline odds and 8-year change in odds of social isolation, living arrangement, core discussion network size, religious services attendance, and social participation by functional hearing, vision, and dual sensory impairment status in independent multivariate generalized logistic mixed models; National Health and Aging Trends Study, 2011-2019^a^

	Baseline			8-year change		
	OR	95% CI	*P*-Value	OR	95% CI	*P*-Value
Social Isolation						
No impairment	1 [Ref.]			1 [Ref.]		
Hearing impairment	0.79	(0.67, 0.92)	0.003	1.28	(1.01, 1.62)	0.044
Vision impairment	1.16	(0.89, 1.51)	0.277	0.96	(0.62, 1.51)	0.876
Dual sensory impairment	1.12	(0.78, 1.62)	0.540	1.22	(0.62, 2.40)	0.561
Domains of Social Isolation		Baseline		8-year change		
Live alone						
No impairment	1 [Ref.]			1 [Ref.]		
Hearing impairment	0.93	(0.80, 1.09)	0.397	1.31	(1.07, 1.59)	0.008
Vision impairment	1.33	(1.02, 1.74)	0.034	0.76	(0.52, 1.11)	0.157
Dual sensory impairment	1.06	(0.72, 1.56)	0.784	1.81	(1.00, 3.26)	0.049
Small core discussion network size						
No impairment	1 [Ref.]			1 [Ref.]		
Hearing impairment	0.93	(0.83, 1.05)	0.268	1.04	(0.84, 1.29)	0.717
Vision impairment	1.19	(0.95, 1.48)	0.130	0.74	(0.48, 1.14)	0.175
Dual sensory impairment	0.95	(0.68, 1.31)	0.740	1.13	(0.57, 2.22)	0.726
No past month religious services attendance						
No impairment	1 [Ref.]			1 [Ref.]		
Hearing impairment	0.83	(0.72, 0.96)	0.010	1.10	(0.93, 1.29)	0.257
Vision impairment	0.99	(0.77, 1.28)	0.967	1.12	(0.81, 1.55)	0.476
Dual sensory impairment	1.09	(0.76, 1.54)	0.649	1.58	(0.96, 2.61)	0.071
No past month social participation						
No impairment	1 [Ref.]			1 [Ref.]		
Hearing impairment	0.79	(0.69, 0.89)	0.000	1.38	(1.14, 1.67)	0.001
Vision impairment	1.02	(0.81, 1.28)	0.885	1.44	(0.97, 2.14)	0.070
Dual sensory impairment	1.19	(0.84, 1.70)	0.321	1.54	(0.80, 2.94)	0.196

^a^Models adjusted for age, sex, education, and race/ethnicity, smoking status, hypertension, diabetes, stroke, heart attack, heart disease, lung disease, and cancer

By domain, vision impairment only (vs. no impairment) was associated with higher baseline odds of living alone (OR: 1.33; 95% CI: 1.02, 1.74), but this difference did not persist over time. No baseline differences were observed by hearing and dual sensory impairment status; but over 8 years, hearing and dual sensory impairments were associated with 31% (OR: 1.31; 95% CI: 1.07, 1.59) and 81% (OR: 1.81; 95% CI: 1.00, 3.26) greater odds of living alone, respectively. No significant differences in core discussion network size were observed by hearing, vision, or dual sensory impairment status. For past month attendance at religious services, hearing impairment only (ref: no impairment) was associated with lower baseline odds of no religious services attendance (OR: 0.83; 95% CI: 0.72, 0.96). No significant differences in religious services attendance over time were observed by hearing impairment only, vision impairment only, and dual sensory impairment status. Finally, for past month social participation, hearing impairment only (vs. no impairment) was associated with lower odds of no social participation at baseline (OR: 0.79; 95% CI: 0.69, 0.89). Over 8 years, hearing impairment only (vs. no impairment) was associated with 38% higher odds of no past month social participation (OR: 1.38; 95% CI: 1.14, 1.67). No significant differences were observed by vision impairment only or dual sensory impairment status; however, the magnitudes of association for change in social participation over time for functional vision (OR: 1.44; 95% CI: 0.97, 2.14) and dual sensory impairment (OR: 1.54; 95% CI: 0.80, 2.94) are nearly the same or larger as observed for hearing impairment only (OR: 1.38; 95% CI: 1.14, 1.67), but confidence intervals were wider potentially due to lower sample size (vison impairment: *n* = 264, dual sensory impairment: *n* = 127).

In a sensitivity analysis, given concerns that change in social isolation over time may be non-linear, we modeled time using a linear spline with one knot at round 5. We observed no significant difference in the magnitude of association over time before and after round 5 by functional sensory impairment status. Thus, primary findings are robust to sensitivity analyses modeling non-linear change in social isolation over time. Additionally, when hearing and vision impairment were assessed independently in single sensory impairment models, results were consistent with primary findings (Supplemental Tables 1 and 2).

## Discussion

Our findings suggest that functional hearing impairment may limit some, but not all, aspects of social structure and engagement among older adults. In a sample of 5,552 community-dwelling older adults in the U.S., 18.9% self-reported hearing impairment only, 4.8% self-reported vision impairment only, and 2.3% self-reported dual sensory impairment only. Hearing impairment only (vs. no impairment) was associated with 28% greater odds of social isolation over 8 years. Specifically, stronger associations were observed with two specific factors of social isolation: living alone and lack of social participation. To our knowledge, this study is one of the first to assess the longitudinal associations between hearing, vision, and dual sensory impairment and social isolation in older adults.

We discuss findings separately by sensory impairment and by factor of social isolation; due to varying definitions and methods of measuring social isolation, associations with social isolation composites may not be comparable across studies. Observed associations between hearing impairment and social isolation are consistent with prior studies. Our finding that older adults with hearing impairment were more likely to live alone over time is consistent with descriptive data from several studies [25–27]. A similar association is also seen in the disability research literature; persons with a disability are twice as likely to live alone and to live alone for longer periods of time [28].

Also consistent with previous findings, we find older adults with hearing impairment continue to remain active in religious services but may restrict social participation (e.g., clubs, volunteer work). In studies of older Europeans, participation in religious services was similar by hearing impairment status; however, older adults with hearing impairment were less likely to participate in other activities such as recreational and sports activities, educational/training courses, or political/community organizations [29, 30]. Older adults may choose to remain active in activities that are more accessible for people with sensory impairment and/or rely less on ability to communicate. For example, accommodations, such as amplification devices, may be more commonly available for religious services than for other activities [31]. Similarly, as older adults with hearing impairment are less likely to drive or take public transportation,[32, 33] activities that provide support for transportation are more accessible to attend. Carpools/assistance with travel may be more likely to be offered for religious services than for other activities.

The literature on hearing impairment and social network size is mixed. Our findings are consistent with Mick et al. and Lind et al. who show no association between hearing impairment and core discussion network size measured by number of close friends and number of important people in their life, respectively [6, 34]. However, Kramer et al. and Ogawa do observe smaller social network size among older adults with hearing impairment [35, 36]. Differences in findings across studies may be due to differences in methods of measuring both hearing impairment and social network size.

In contrast to prior research, we did not observe differences in any measures of social isolation across time by vision impairment status [7–10]. While findings for functional vision impairment from the current study are not statistically significant, the patterns of point estimates are informative. Consistent with potential ceiling effects, point estimates show higher likelihood of social isolation at baseline but minimal differences across time among participants with functional vision impairment compared to no impairment. Changes in social structure and engagement associated with functional vision impairment may have occurred prior to the study period, reflected by elevated baseline odds. Thus, at the start of the study, level of social isolation may have already reached its maximum and, as a result, even greater increases in social isolation over the study period were not observed. One explanation for these findings is that because vision impairment may exert greater negative impact on mobility and participation in activities than hearing impairment [7, 37], restrictions in social interactions and activity may occur earlier and more immediately following onset of vision impairment and thus were not captured in our study period. Future research should further explore these findings.

Greater likelihood of social isolation over time was also observed among older adults with dual sensory impairment, but some estimates lacked precision. Dual sensory impairment was significantly associated with greater odds of living alone over time, consistent with descriptive data from prior studies [25, 26]. Additionally, point estimates show greater odds of no past month attendance at religious services and no past month social participation at baseline and across time among participants with dual sensory impairment, but estimates lacked precision and did not reach the threshold for statistical significance likely given low sample size. Even if not statistically significant, this pattern of results suggests a negative impact of functional dual sensory impairment on social engagement and participation.

Previous studies have also observed associations between dual sensory impairment and social isolation [7, 29, 30]. For example, among individuals 65–85 years in the Canadian Longitudinal Study on Aging, dual sensory impairment was associated with reduced social participation and lower availability of social support [7]. Additionally, data from the Survey of Health, Ageing and Retirement in Europe and the German Ageing Survey also find higher social isolation and social inactivity among participants with dual sensory impairment, but associations were attenuated after adjustment for health and socioeconomic factors [29, 30]. Older adults with dual sensory impairment may be a subgroup particularly at risk for social isolation because it can be more difficult to employ compensation mechanisms to maintain functional ability. In single sensory impairment, individuals can use one sense to compensate for loss of function in another sense (sensory substitution); however, this is more difficult in dual sensory impairment. As a result, maintaining mobility and the ability to communicate and navigate social situations may be more challenging for older adults with dual sensory impairment [18] and can lead to fewer opportunities for social connection [38].

### Limitations

First, this study assesses structural social isolation. While we were unable to comment on other aspects of social isolation such as quality of social relationships, social support, and loneliness with this sample, the typology of structural social isolation used in this study is advantageous. It is informed by the Berkman-Syme Social Network Index and Lubben Social Network Scale, incorporating multiple domains of social connection (living arrangement, core discussion network size, religious services attendance, and social participation) [5]. Second, we were unable to capture characteristics of the environments in which participants live; those living in more accessible environments may have more opportunities for social connection. Additionally, it is possible that our findings underestimated the true association between sensory impairment and social isolation due to greater study attrition over time among participants who have functional sensory impairment and who have experienced social isolation. Further, older adults with sensory impairment may be less likely to enroll in this study given concerns about their ability to complete tasks included in the data collection. Greater uncertainty around our findings regarding dual sensory impairment may be because our cohort was underpowered to detect a statistically significant association given the low number of participants with dual sensory impairment at baseline (*n* = 127). Finally, findings are not representative of the 4% of older adults in the U.S. not enrolled in Medicare. The analytic sample also excludes participants who lived in nursing home/residential facilities and who met the empirical criteria for dementia at baseline. Thus, findings are generalizable to cognitively healthy, community-dwelling Medicare beneficiaries.

### Implications

In light of the COVID-19 pandemic, social isolation has been brought to the forefront of public health [39]. Clinicians, policymakers, and researchers are increasingly interested in understanding the potentially debilitating impact of social isolation on health and opportunities to mitigate its effect. Improved awareness of subgroups most at risk for social isolation is necessary. Clinically, self-reported questions of sensory loss are quick, easy to administer, and have strong value for inclusion in clinical settings [40] to help identify older adults who may benefit from a discussion of their social connection, referral to community-based resources aiming to address social isolation, such as senior companion/care programs [1, 41], or social prescribing. Additionally, clinical referral for sensory aids and treatment may also be beneficial as sensory aids can improve communication and could potentially reduce social isolation. [42] This work supports policy efforts advocating for greater affordable access to high quality vision and hearing care and sensory aids through expansion of Medicare coverage to include care and treatment of hearing and vision impairments. Furthermore, integrating universal design (designing products and the environment to be accessible for all people with a wide range of abilities, disabilities, and characteristics) as a societal norm will increase accessibility for older adults with sensory impairment in all areas of life [43]. For example, universal design integrated into public places (e.g., restaurants, public transportation) can help older adults safely participate in activities outside the home and increase opportunities for social connection [43]. Similarly, creating technology with integrated low-vision and low-hearing accessibility may allow older adults with sensory impairments to more easily connect with others virtually. Finally, this work also informs future research of other health outcomes associated with sensory impairment (e.g., cognitive decline and dementia, depression) where social isolation may act as a potential mediator of these relationships.

In an 8-year longitudinal cohort study of older adults in the U.S., self-reported hearing impairment only was associated with higher odds of social isolation over time. Older adults with hearing impairment may benefit from interventions aiming to increase social connection as well as optimization of the design of environments to become more universally accessible to older adults with sensory impairment. Future investigations may also consider treatment for hearing impairment and rehabilitation strategies to aid in increasing social connection among older adults with hearing impairment.

### Supplementary Information

**Additional file 1: Supplemental Table 1. **Adjusted baseline odds and 8-year change in odds of social isolation, living arrangement, core discussion network size, religious services attendance, and social participation by functional hearing impairment status in independent multivariate generalized logistic mixed models; National Health and Aging Trends Study, 2011-2019^a^. **Supplemental Table 2.** Adjusted baseline odds and 8-year change in odds of social isolation, living arrangement, core discussion network size, religious services attendance, and social participation by functional vision impairment status in independent multivariate generalized logistic mixed models; National Health and Aging Trends Study, 2011-2019^a^. 

## Data Availability

The datasets analyzed in the current study are publicly available at www.nhats.org.

## Abbreviation

NHATS: National Health and Aging Trends Study
