# Supplementary material for: Hearing and vision impairment and social isolation over 8 years in community-dwelling older adults
Source: BMC Public Health. 2024 Mar 13;24:779. doi: 10.1186/s12889-024-17730-8 (PMC10936068; doi:10.1186/s12889-024-17730-8)
Supplement: Supplementary file 1 — Additional file 1: Supplemental Table 1. Adjusted baseline odds and 8-year change in odds of social isolation, living arrangement, core discussion network size, religious services attendance, and social participation by functional hearing impairment status in independent multivariate generalized logistic mixed models; National Health and Aging Trends Study, 2011-2019a. Supplemental Table 2. Adjusted baseline odds and 8-year change in odds of social isolation, living arrangement, core discussion network size, religious services attendance, and social participation by functional vision impairment status in independent multivariate generalized logistic mixed models; National Health and Aging Trends Study, 2011-2019a. [file 12889_2024_17730_MOESM1_ESM.docx]

**Supplemental Tables**

Supplemental Table 1. Adjusted baseline odds and 8-year change in odds of social isolation, living arrangement, core discussion network size, religious services attendance, and social participation by functional hearing impairment status in independent multivariate generalized logistic mixed models; National Health and Aging Trends Study, 2011-2019^a^

|  | Baseline | | | 8-Year Change | | |
| --- | --- | --- | --- | --- | --- | --- |
|  | OR | 95% CI | P-Value | OR | 95% CI | P-Value |
| Social Isolation |  |  |  |  |  |  |
| No hearing impairment | 1 [Ref.] | | | 1 [Ref.] | | |
| Hearing impairment | 0.81 | (0.70, 0.94) | 0.006 | 1.27 | (1.01, 1.59) | 0.041 |
| Domains of Social Isolation |  |  |  |  |  |  |
| Live alone |  |  |  |  |  |  |
| No hearing impairment | 1 [Ref.] | | | 1 [Ref.] | | |
| Hearing impairment | 0.93 | (0.80, 1.08) | 0.324 | 1.36 | (1.13, 1.64) | 0.001 |
| Small core discussion network size |  |  |  |  |  |  |
| No hearing impairment | 1 [Ref.] | | | 1 [Ref.] | | |
| Hearing impairment | 0.93 | (0.82, 1.04) | 0.191 | 1.06 | (0.86, 1.31) | 0.566 |
| No past month religious services attendance |  |  |  |  |  |  |
| No hearing impairment | 1 [Ref.] | | | 1 [Ref.] | | |
| Hearing impairment | 0.86 | (0.75, 0.98) | 0.023 | 1.12 | (0.96, 1.31) | 0.148 |
| No past month social participation |  |  |  |  |  |  |
| No hearing impairment | 1 [Ref.] | | | 1 [Ref.] | | |
| Hearing impairment | 0.82 | (0.72, 0.92) | 0.001 | 1.36 | (1.13, 1.63) | 0.001 |

^a^ Models adjusted for age, sex, education, and race/ethnicity, smoking status, hypertension, diabetes, stroke, heart attack, heart disease, lung disease, and cancer

Supplemental Table 2. Adjusted baseline odds and 8-year change in odds of social isolation, living arrangement, core discussion network size, religious services attendance, and social participation by functional vision impairment status in independent multivariate generalized logistic mixed models; National Health and Aging Trends Study, 2011-2019^a^

|  | Baseline | | | 8-year change | | |
| --- | --- | --- | --- | --- | --- | --- |
|  | OR | 95% CI | P-Value | OR | 95% CI | P-Value |
| Social Isolation |  |  |  |  |  |  |
| No vision impairment | 1 [Ref.] | | | 1 [Ref.] | | |
| Vision impairment | 1.21 | (0.98, 1.51) | 0.081 | 0.98 | (0.68, 1.43) | 0.929 |
| Domains of Social Isolation |  |  |  |  |  |  |
| Live alone |  |  |  |  |  |  |
| No vision impairment | 1 [Ref.] | | | 1 [Ref.] | | |
| Vision impairment | 1.26 | (1.01, 1.58) | 0.041 | 0.93 | (0.67, 1.28) | 0.641 |
| Small core discussion network size |  |  |  |  |  |  |
| No vision impairment | 1 [Ref.] | | | 1 [Ref.] | | |
| Vision impairment | 1.13 | (0.94, 1.35) | 0.211 | 0.83 | (0.57, 1.20) | 0.314 |
| No past month religious services attendance |  |  |  |  |  |  |
| No vision impairment | 1 [Ref.] | | | 1 [Ref.] | | |
| Vision impairment | 1.07 | (0.87, 1.32) | 0.525 | 1.22 | (0.93, 1.60) | 0.153 |
| No past month social participation |  |  |  |  |  |  |
| No vision impairment | 1 [Ref.] | | | 1 [Ref.] | | |
| Vision impairment | 1.13 | (0.93, 1.37) | 0.235 | 1.37 | (0.98, 1.93) | 0.069 |

^a^ Models adjusted for age, sex, education, and race/ethnicity, smoking status, hypertension, diabetes, stroke, heart attack, heart disease, lung disease, and cancer
